# Assessing the Benefits of an Innovative Chemical Peel Containing Biofunctionals on Adult Acne‐Prone Skin: An Exploratory Interventional Study: A Preliminary Report

**DOI:** 10.1111/jocd.70772

**Published:** 2026-03-05

**Authors:** Sarah Brooks, Savitha Rajagopal, Marc Z. Handler, Mark Vandeven, Angela Carlile

**Affiliations:** ^1^ Research and Development Colgate‐Palmolive Company, PCA SKIN Scottsdale Arizona USA; ^2^ Department of Dermatology Rutgers New Jersey Medical School Newark New Jersey USA; ^3^ Dermatology Hackensack Meridian School of Medicine Nutley New Jersey USA; ^4^ Colgate Palmolive Company Piscataway New Jersey USA

**Keywords:** acne vulgaris, chemical peel, sebum, skin clarity, skin texture, small‐scale study

## Abstract

**Background:**

Acne is the most prevalent skin disorder in the United States, affecting up to 50 million people from all age groups. Treatment options include topical and systemic therapies. Limitation in many treatment options opens avenues for alternative therapies, such as chemical peeling.

**Aims:**

This exploratory study, funded by Colgate Palmolive company, aimed to evaluate the effectiveness of a new chemical peel (PCAskin Acne Peel Plus) in treating adult acne. The novel peel features a blend of acids and biofunctional ingredients designed to aid in acne management. The study's primary objective was to assess the novel peel's effectiveness in positively influencing acne severity, specifically by reducing acne lesions, papules, and pustules.

**Patients:**

Sixteen participants aged 25–40 years old, with Fitzpatrick skin types I–VI, presenting evidence of mild‐to‐moderate acne, were assessed over a 12‐week period following treatment initiation.

**Evaluation Methods:**

The effects of the test peel on acne were evaluated using a combination of methods. Skin sebum was measured using a moisture meter (BGJOY, SK‐IV digital moisture monitor for skin). Photographic data was obtained using the Canfield Visia CR System (Canfield, Fairfield, NJ; model Generation 7, software version 8) for determining acne severity, appearance of skin pores, texture and redness. Acne severity was assessed by the study investigator using the Investigator Global Assessment (IGA) acne severity scale from the Visia images. Subjective assessment of skin parameters (acne severity, oiliness of the skin, pore size, skin discoloration, skin texture/smoothness, overall clarity of skin tone, and changes in scarring appearance) was also obtained at the start (Day 0) and end (Week 12) of the study using self‐assessment questionnaires filled out by the study subjects.

**Results:**

Significant decreases in total acne lesions (papules + pustules; *p*‐value = 0.012) and papules (*p*‐value = 0.023) were observed at the outset of the study (Week 12) compared to baseline (Day 0), with an average change (standard deviation) of −2.0 (2.6) and −1.8 (2.5) lesions, respectively. In addition, significant improvements in sebum content (Week 12, *p*‐value = 0.042), erythema (Week 12, *p*‐value = 0.030), and pore appearance (Day 1; *p*‐value = 0.005; Week 12, *p*‐value = 0.003) were observed compared to baseline. Positive perceptions of the treatment among participants and perceived improvements in acne severity (*p*‐value = 0.004) and skin clarity (*p*‐value = 0.036) were also highlighted. No adverse effects were observed during the study.

**Conclusions:**

This preliminary exploratory study indicates that treatment with a novel chemical peel appeared to yield a range of benefits for adults with acne‐prone skin, supporting its potential as a safe, inexpensive, and minimally invasive treatment for the management of mild‐to‐moderate forms of adult acne. Further large scale, controlled studies are necessary to confirm these initial findings.

## 1 Introduction

Acne vulgaris is one of the most widespread inflammatory diseases in the world [1]. This skin condition is typically characterized by the appearance of comedones, papules, pustules, and nodules [2] on the face, neck, chest, upper back, and upper arm area, all of which may scar upon healing [3]. Although acne is typically associated with young people, particularly those of the 10–19 year old group, a significant number of patients either continue to experience acne or develop new‐onset acne after adolescence [4]. Indeed, it is estimated that close to 10% of acne cases occur in adults older than 25 years of age [5].

Adolescent, or young person acne, and adult acne differ in both their clinical presentation and epidemiology [4]. Adult acne tends to rarely feature comedones and be more inflammatory in nature than adolescent acne, which generally starts with comedones and later develops into a range of acneiform lesions [4]. The T‐zone (forehead, nose, and cheeks) is affected by both adult and adolescent acne; however, adult acne also presents important mandibular involvement [6]. Conversely, truncal involvement appears to be rarer in adult patients [6]. With regards to epidemiology, adolescent acne is more common in the male than the female population, with epidemiological data indicating that 27.9% of boys and 20.8% of girls are affected by adolescent acne [4]. Conversely, adult acne appears to be more prevalent in women, with an epidemiological study reporting that 82.1% of participants with adult acne are women [6]. Another study highlighted reports of late‐onset acne (acne developing for the first time after the age of 25, as opposed to persistent acne, which starts in adolescence and continues into adulthood, or relapsing acne, which heals and reappears in episodes throughout both adolescence and adulthood) in 97.3% of women [7]. Finally, scarring appears to be more common as a result of adult acne than adolescent acne, with reports that 20%–76.4% of people with adult acne developed some form of scarring [4, 5, 6].

While acne is not a life‐threatening disease, it has a consequent impact on appearance, and its clinical presentation and associated scarring involve a heavy psychosocial burden comparable to chronic systemic diseases such as asthma, epilepsy, diabetes, and arthritis, with some people affected by acne experiencing depression, anxiety, and lower self‐esteem [8, 9]. An observational study showed significantly higher Hospital Anxiety and Depression Scale scores for anxiety and depression for people with acne compared to a control group, with 40.6% reporting serious concern about the disease and 4% who considered that acne was the underlying cause of suicidal ideation [10]. Significantly more important decreases in self‐esteem were reported from women than men affected by acne [11]. In fact, most studies on the subject reported that women with acne were at increased risk of experiencing higher self‐consciousness and self‐perceived stress, lower levels of self‐esteem and self‐worth, lower levels of body satisfaction, lower levels of self‐attitude, and higher levels of feelings of helplessness than men with acne [12, 13].

In light of the burden of disease and potential sequelae, the implementation of prompt and effective treatment is critical. Common treatments for acne include topical treatments (e.g., retinoids, benzoyl peroxide), systemic treatments (e.g., tetracycline antibiotics), or a combination of both topical and systemic therapies [4]. In addition to traditional therapy, other treatments such as laser therapy, phototherapy, and chemical peeling have been reported to promote faster recovery from active lesions and decrease the risk of scarring [14]. Chemical peeling promotes skin regeneration and remodeling of the tissue via controlled chemical‐induced injury to the skin and is considered to be a safe, inexpensive professional treatment/non‐invasive treatment for acne [15].

The present study aimed to assess the benefits of a novel, commercially available chemical peel, containing a combination of hydroxy acids along with biofunctional ingredients that work together to achieve optimal skin exfoliation, sebum control, acne management, antioxidant, and anti‐inflammatory action. The blend of lactic acid and salicylic acids works together to remove surface debris and decongest clogged pores, while resorcinol helps loosen dead skin cells in acne‐prone skin. On the other hand, oleyl adapalenate, a next generation retinoid, prevents inflamed acne lesions from forming. Antioxidant and anti‐inflammatory blend (saccharide isomare, licorice root extract and lipoic acid) acts as a calming and soothing blend that reduces visible redness and discoloration in an acne‐affected skin.

The blend was evaluated in people aged ≥ 25 years old, with acne‐prone skin. To this end, reduction of acne lesions (as evaluated by the study dermatologist), skin parameters contributing to acne, namely sebum production, other skin parameters (dilated pores, skin smoothness, erythema/skin redness), and the participants' self‐assessment of treatment response were evaluated over a 12‐week period after treatment initiation.

## 2 Materials and Methods

### 2.1 Study Design

This small‐scale, unblinded, exploratory study aimed to assess the efficacy and benefits of a commercially available chemical peel (*Acne Peel Plus Advanced*, PCA SKIN, Colgate Palmolive skin health group, Scottsdale, AZ) in adults with acne‐prone skin. The study protocol was approved by the United States Institutional Review Boards (U.S. IRB) on the 3rd of October 2023 (PCA‐2023‐600, USIRB2023CPS/04). This exploratory in vivo study was conducted to ascertain the benefits of a novel peel and is not a registered clinical trial. We emphasize that this is a preliminary, exploratory study for cosmetic products under US regulations, not a clinical trial, and therefore, clinical trial registration is not mandatory. The study's objective is to understand the top‐line benefits of the proprietary product, which is neither a drug nor a medical device, and is thus not subject to FDA mandates requiring benefit demonstration through a clinical trial. The study was conducted by a trained aesthetician at the PCA SKIN corporate headquarters in Scottsdale, Arizona, in the period from October 2023 to March 2024.

The study was carried out over 12 weeks, with participants receiving the chemical peel at Day 0, Week 4, and Week 8. Photographic data and sebum production measurements were collected prior to treatment initiation (baseline; Day 0), one day after treatment initiation (Day 1), as well as at Week 4, Week 8, and Week 12. The participants were also tasked with completing a self‐assessment questionnaire at the start (Day 0) and end (Week 12) of the study.

### 2.2 Study Participants

Sixteen participants (male or female) aged 25–40 years old with Fitzpatrick skin types I–VI, presenting evidence of mild‐to‐moderate acne were enrolled in the study. Most medical professionals recommend chemical peels for individuals in their early twenties. For this study, due to the peel's strength and new formulation, we conservatively targeted adults prone to acne aged 25, rather than younger subjects (e.g., 18 years) with similar skin concerns. Acne severity was assessed by the study investigator (dermatologist) using the Investigator Global Assessment (IGA) acne severity scale [16] and only subjects with grade 2 (mild severity; some noninflammatory lesions with no more than a few inflammatory lesions [papules/pustules only, no nodular lesions]) and grade 3 (moderate severity; up to many noninflammatory lesions and may have some inflammatory lesions, but no more than one small nodular lesion) were included in the study.

Participants were asked to limit excessive ultraviolet light exposure and other behaviors that may affect study results (e.g., use of skin products unrelated to the study, cosmetic procedures affecting the face, smoking and vaping). Pregnant or breastfeeding women, individuals with known allergies to ingredients of the chemical peel treatment or support products, and individuals treated with systemic oral retinoids, oral contraceptives, or topical treatments for acne were excluded from the study. A full list of inclusion and exclusion criteria is available in Supporting Information S1.

The treatment procedure was explained to all the participants in detail, and verbal and informed written consent was obtained prior to the start of the treatment procedure.

### 2.3 Treatment Procedure

The chemical peel tested here contains a combination of several acids (e.g., lactic acid, salicylic acid, thioctic acid) and compounds included to promote skin regeneration following chemically induced exfoliation (e.g., resorcinol, oleyl adapalenate, licorice root extract, saccharide isomerate) composition as presented in Table 7.

Before starting the treatment procedure, participants observed a 14‐day pre‐study washout period. During this time, they stopped using retinoids and exclusively used PCA SKIN Creamy Cleanser, PCA SKIN Hydrator Plus Broad Spectrum SPF 30, and PCA SKIN ReBalance moisturizer for their facial skincare. This 2‐week washout period is standard (typically 1–4 weeks) [17], striking a balance between eliminating effects from previous products and preventing extended treatment breaks that could worsen patient acne. The study support products were free of active ingredients that might influence the study's results.

At the established treatment time points (Day 0, Week 4, and Week 8), prior to treatment administration, the participants' facial skin was cleansed twice with the PCA SKIN Creamy Cleanser and prepared with the PCA SKIN Smoothing Toner. The chemical peel treatment was then applied to the face in layers by the study investigator. Skin sensitivity was gauged after application of each layer using an internal use 1–10 sensitivity scale, and a second layer of the experimental treatment was applied if the participant scored 5 or lower. In this study, all the participants were able to tolerate 2 layers.

Participants were provided with study support products to take home and use for the morning and night routines over the course of the study. The morning routine consisted of facial cleansing with the PCA SKIN Creamy Cleanser and application of the PCA SKIN Hydrator Plus Broad Spectrum SPF 30 sunscreen product. The night routine consisted of facial cleansing with the PCA SKIN Creamy Cleanser and application of the PCA SKIN ReBalance moisturizer. As per study instructions, the participants were to use no other facial skincare products during the 14‐day pre‐study washout and 12‐week study periods.

### 2.4 Evaluations

Photographic data was obtained using the Canfield Visia CR System (Canfield, Fairfield, NJ; model Generation 7, software version 8). For each participant, images of both left and right sides of the face, as well as a frontal view were obtained at each time point. Standard lighting was used to assess visible features (e.g., spots, pores, and texture) and cross‐polarized and UV lighting was used to detect subsurface details (e.g., erythema). Collected images were used for (i) the evaluation of acne lesions (pustules and papules) at Day 0 and Week 12 by the study investigator who is a qualified dermatologist and (ii) automated quantification of skin parameters (dilated pores, skin smoothness, and erythema/skin redness) using the VISIA skin analysis system.

Sebum production was evaluated using Bio‐instruments moisture meter (BGJOY, SK‐IV digital moisture monitor for skin). The same sites on the forehead were used for measurements throughout the study. At each time point, three measurements were taken, the average of which was used for analyses.

Subjective assessment of skin parameters was also obtained at the start (Day 0) and end (Week 12) of the study using self‐assessment questionnaires completed by the participants. Participants were asked to rate acne severity, oiliness of the skin, pore size, skin discoloration, skin texture/smoothness, overall clarity of skin tone, and changes in scarring appearance on a scale from 0 to 4. Questions appearing in the Week 0 and Week 12 self‐assessment questionnaires are listed in Supporting Information S2.

Statistical analysis of the data was performed using the Wilcoxon signed rank test. Statistical significance was defined as a *p*‐value ≤ 0.05.

### 2.5 External Monitoring/Quality Control

This preliminary exploratory study was conducted in the PCASkin Scottsdale Arizona campus, and the team partnered with Colgate Palmolive's Global Technology Center in Piscataway, New Jersey. An external dermatologist, based in New Jersey, did the assessment independent of the study using Visia images.

## 3 Results

### 3.1 Acne Severity

Acne severity was evaluated by an independent consultant dermatologist using the Investigator Global Assessment (IGA) [16] scale, a five‐point grading system recommended by the FDA and widely recognized by dermatologists. Operating outside the study site and not involved in treatment, this blinded consultant professionally assessed the “Before and After” images. The consultant applied the universal IGA scale to quantify changes in acne severity, thereby grading the effective skin change based on this standardized measure.

**FIGURE 1:**
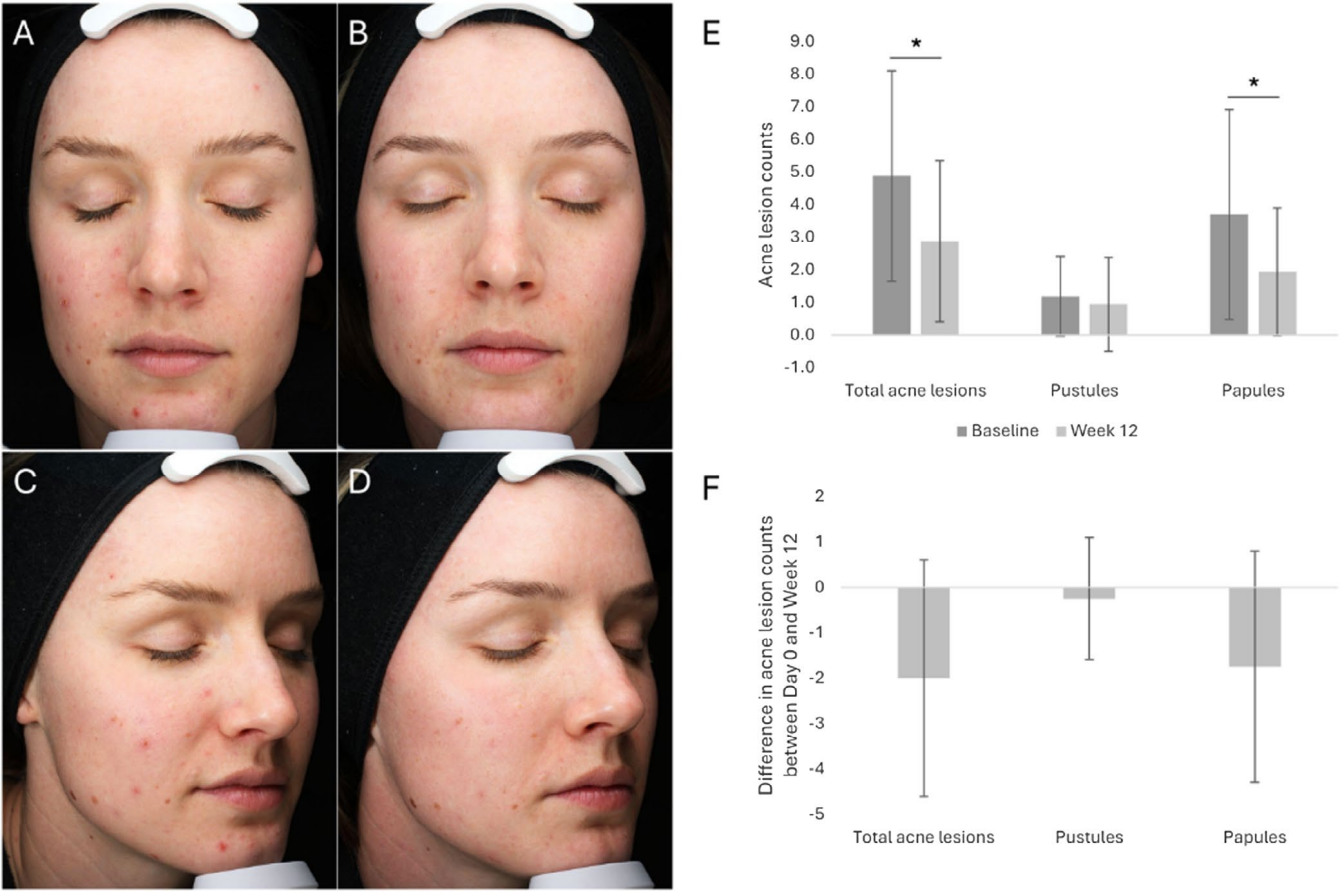
Acne severity. (A–D) Representative photographic images (standard lighting) prior to the initiation of the treatment (Day 0; panels A and C) and at Week 12 after treatment initiation (panels B and D). (E) Average counts of total acne lesions (pustules + papules), pustules, and papules prior to the initiation of the treatment (Baseline; Day 0) and at Week 12 after treatment initiation. (F) Average relative change in counts of total acne lesions, pustules, and papules between baseline (Day 0) and Week 12. The error bars correspond to the standard deviation. *p‐value ≤ 0.05 is considered statistically significant. *n*, number of participants.

Assessments were conducted by the study dermatologist at the start of treatment (Day 0) and at the end of the 12‐week study period. The study observed a positive trend, with overall decreases in total acne lesions, pustules, and papules. At baseline, the average counts were 4.9 for total acne lesions, 1.2 for pustules, and 3.7 for papules. By Week 12, these counts had reduced to 2.9, 0.9, and 1.9, respectively (Table 1, Figure 1A–F). These reductions correspond to a 41.0% decrease in total acne lesions, a 21.1% decrease in pustules, and a 47.5% decrease in papules. On average, participants experienced 2.0 fewer total acne lesions, 0.3 fewer pustules, and 1.8 fewer papules after completing the treatment when compared to baseline counts (Table 1, Figure 1A–F). Statistical analysis revealed significant decreases in total acne lesions (*p*‐value = 0.012) and papules (*p*‐value = 0.023). However, the decrease in pustules was not statistically significant (*p*‐value = 0.594) (Table 1, Figure 1A–F).

**TABLE 1:** Average lesion counts and average relative change in lesion counts between baseline (Day 0) and Week 12 for total acne lesions (pustules + papules), pustules, and papules.

Time point, number of participants (*n*)	Absolute number		Change from baseline
	Average (SD)	*p*	Average (SD)
Total acne lesions			
Baseline (Day 0), *n* = 16	4.9 (3.2)		
Week 12, *n* = 16	2.9 (2.5)	0.012*	−2.0 (2.6)
Pustules			
Baseline (Day 0), *n* = 16	1.2 (1.2)		
Week 12, *n* = 16	0.9 (1.4)	0.594	−0.3 (1.3)
Papules			
Baseline (Day 0), *n* = 16	3.7 (3.2)		
Week 12, *n* = 16	1.9 (1.9)	0.023*	−1.8 (2.5)

Abbreviation: SD, standard deviation.

* *p*‐value ≤ 0.05 is considered statistically significant.

### 3.2 Sebum Production

**FIGURE 2:**
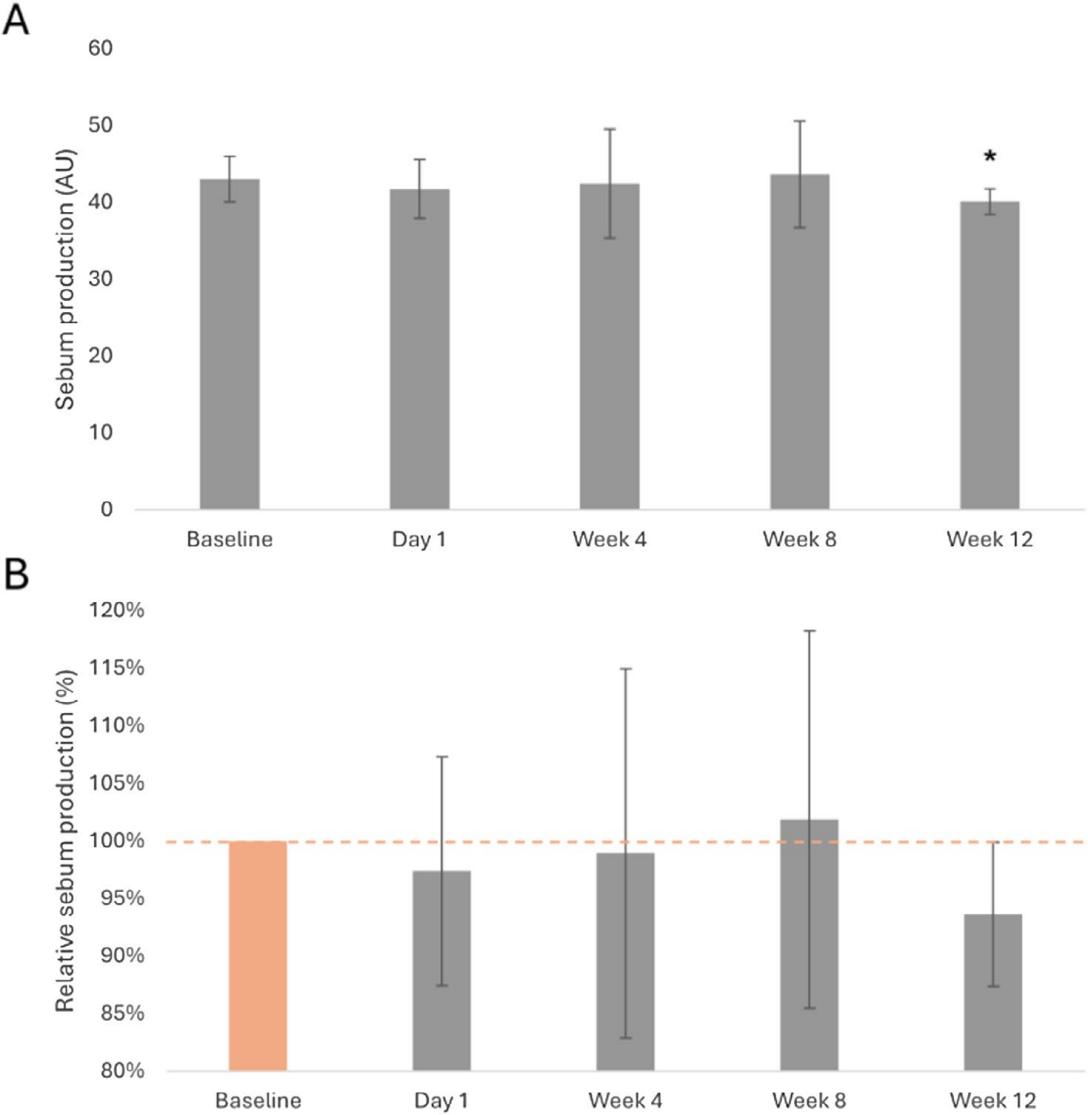
Facial sebum production. (A) Average sebum production measurements prior to the initiation of the treatment (Baseline; Day 0), and at Day 1, Week 4, Week 8, and Week 12 after treatment initiation. (B) Average relative sebum production at baseline (Day 0; set at 100%) and Day 1, Week 4, Week 8, and Week 12 after treatment initiation. The error bars correspond to the standard deviation. *p‐value ≤ 0.05 is considered statistically significant. AU, arbitrary unit.

Facial sebum production, assessed via Bio‐instruments moisture meter, appeared stable the day after the treatment, as well as at Week 4 and Week 8, with average relative values ranging from 97.4% to 101.9% of the baseline condition (average relative value compared to baseline [set at 100%]: Day 1, 97.4%; Week 4, 98.9%; Week 8, 101.9%; Table 2, Figure 2A,B). At Week 12, sebum production measurements decreased by 6.4% compared to the baseline condition (average relative value compared to baseline: 93.6%; Table 2, Figure 2A,B). Only Week 12 measurements were significantly different from the baseline condition (*p*‐value = 0.042; Table 2, Figure 2A,B).

**TABLE 2:** Average sebum production measurements and average sebum production relative to baseline (Day 0; set at 100%).

Time point, number of participants (*n*)	Absolute number (AU)		Percent change from baseline (%)
	Average (SD)	*p*	Average (SD)
Baseline (Day 0), *n* = 8	43.0 (3.0)		100.0
Day 1, *n* = 8	41.7 (3.8)	0.441	97.4 (9.9)
Week 4, *n* = 8	42.4 (7.1)	1.000	98.9 (15.0)
Week 8, *n* = 8	43.6 (7.0)	0.834	101.9 (16.4)
Week 12, *n* = 8	40.1 (17)	0.042*	93.6 (6.3)

Abbreviations: AU, arbitrary unit; SD, standard deviation.

* *p*‐value ≤ 0.05 is considered statistically significant.

### 3.3 Facial Skin Parameters

Quantification of dilated pores, skin smoothness, and skin redness was performed by analysis of the images obtained prior to the initiation of the treatment (Day 0) and at Day 1, Week 4, Week 8, and Week 12 after treatment initiation.

**FIGURE 3:**
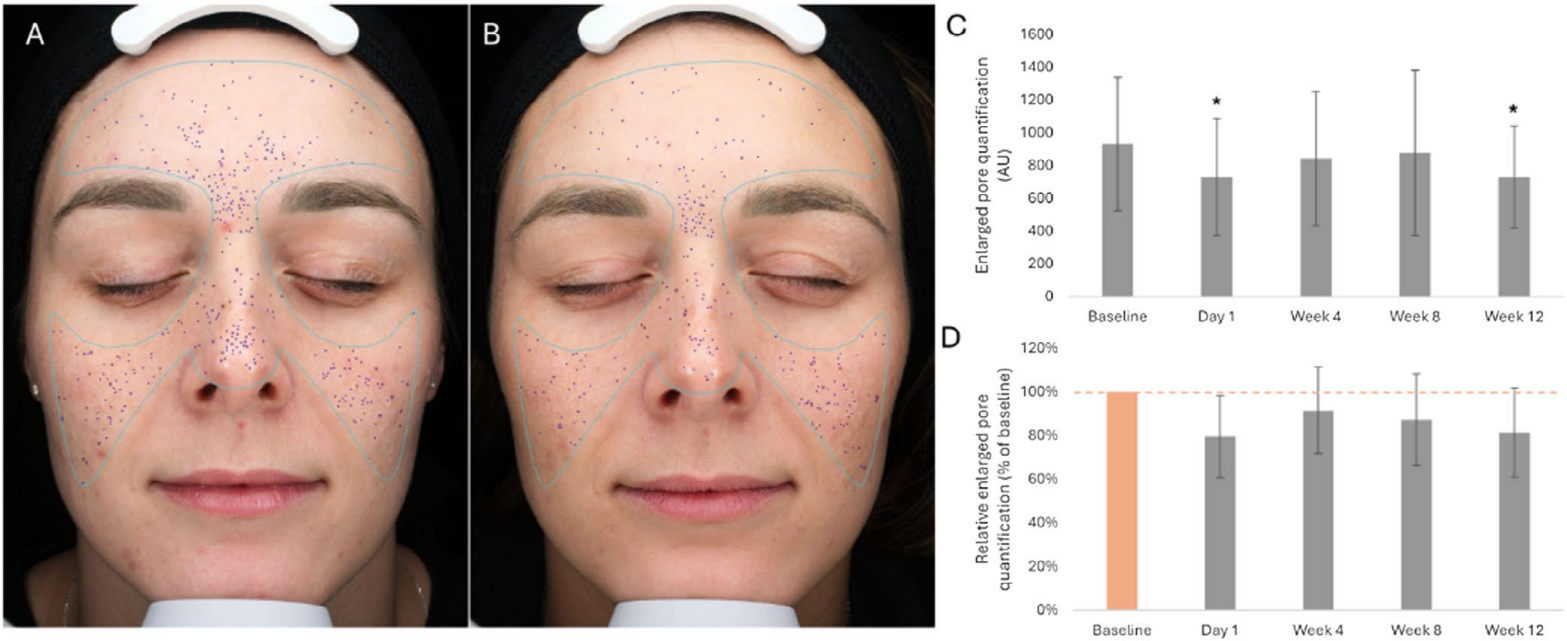
Pore appearance. (A, B) Representative photographic images (standard lighting; VISIA “dilated pore” filter) prior to the initiation of the treatment (Day 0; panels A) and at Week 12 after treatment initiation (panels B). (C) Average of dilated pore quantifications prior to the initiation of the treatment (Baseline; Day 0), and at Day 1, Week 4, Week 8, and Week 12 after treatment initiation. (D) Average relative dilated pore quantifications at baseline (Day 0; set at 100%) and Day 1, Week 4, Week 8, and Week 12 after treatment initiation. The error bars correspond to the standard deviation. *p‐value ≤ 0.05 is considered statistically significant. AU, arbitrary unit.

Average relative quantifications of dilated pores were lower than the baseline condition at all subsequent time points (average relative value compared to baseline [set at 100%]: Day 1, 79.4%; Week 4, 91.5%; Week 8, 87.3%; Week 12, 81.5%; Table 3, Figure 3A–D). Statistical analysis evidenced significant differences in average dilated pore quantifications at Day 1 (*p*‐value = 0.005) and Week 12 (*p*‐value = 0.003) compared to the baseline condition (Table 3, Figure 3A–D); the difference between the Week 4 and baseline, and Week 8 and baseline conditions was not statistically significant (Week 4, *p*‐value = 0.052; Week 8, *p*‐value = 0.118; Table 3, Figure 3A–D).

**TABLE 3:** Average dilated pore quantifications and average dilated pore quantifications relative to baseline (Day 0; set at 100%).

Time point, number of participants (*n*)	Absolute number (AU)		Percent change from baseline (%)
	Average (SD)	*p*	Average (SD)
Baseline (Day 0), *n* = 16	932.2 (408.8)		100.0
Day 1, *n* = 16	729.3 (358.4)	0.005*	79.4 (18.8)
Week 4, *n* = 16	843.2 (409.3)	0.052	91.5 (19.7)
Week 8, *n* = 15	877.1 (504.1)	0.118	87.3 (20.9)
Week 12, *n* = 16	731.0 (310.9)	0.003*	81.5 (20.4)

Abbreviations: AU, arbitrary unit; SD, standard deviation.

* *p*‐value ≤ 0.05 is considered statistically significant.

**FIGURE 4:**
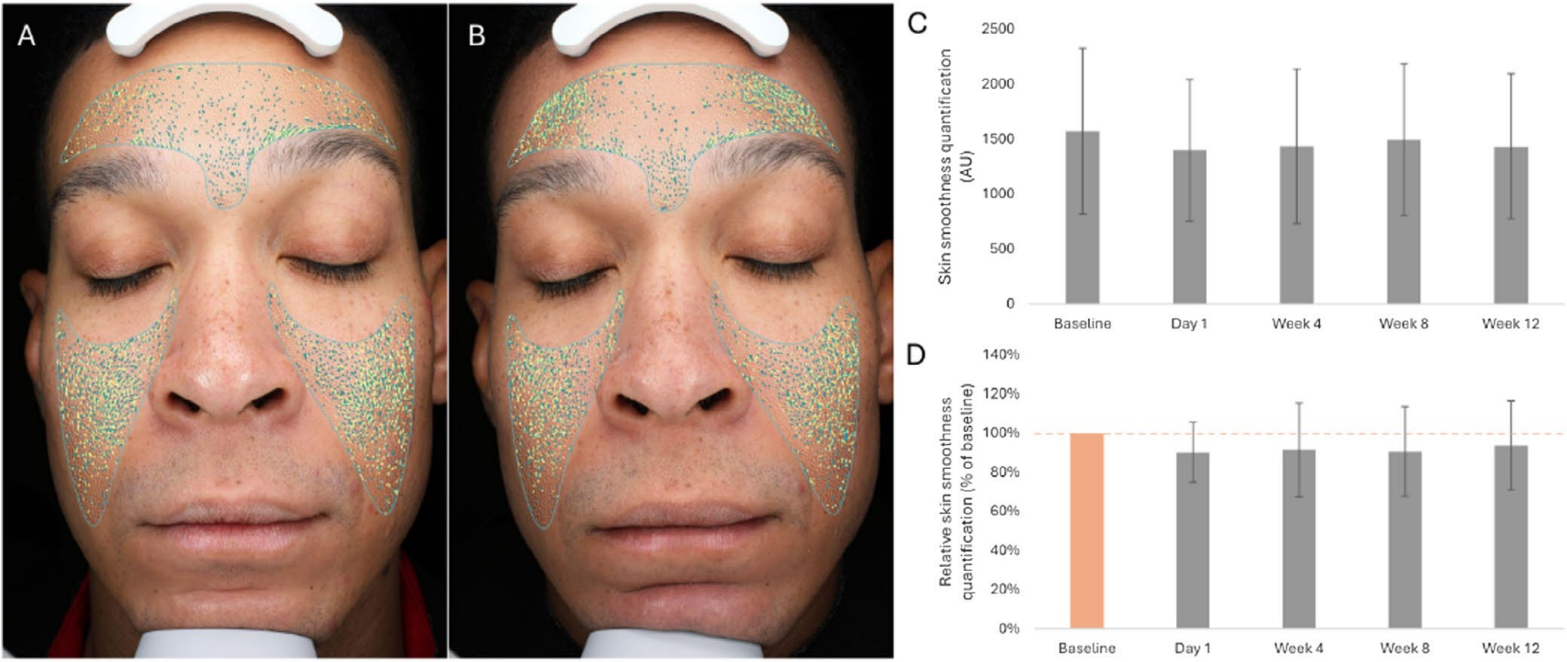
Skin smoothness. (A, B) Representative photographic images (standard lighting; VISIA “skin texture” filter) prior to the initiation of the treatment (Day 0; panels A) and at Week 12 after treatment initiation (panels B). (C) Average of skin smoothness quantifications prior to the initiation of the treatment (Baseline; Day 0), and at Day 1, Week 4, Week 8, and Week 12 after treatment initiation. (D) Average relative skin smoothness quantifications at baseline (Day 0; set at 100%) and Day 1, Week 4, Week 8, and Week 12 after treatment initiation. The error bars correspond to the standard deviation. AU, arbitrary unit.

Average relative quantifications of skin smoothness post‐treatment initiation remained within the 90.1%–93.7% range relative to the baseline condition (average relative value compared to baseline [set at 100%]: Day 1, 90.1%; Week 4, 91.4%; Week 8, 90.7%; Week 12, 93.6%; Table 4, Figure 4). Statistical analysis highlighted no significant differences in average quantifications of skin smoothness between the baseline measurements and post‐treatment initiation time point measurements (Day 1, *p*‐value = 0.059; Week 4, *p*‐value = 0.184; Week 8, *p*‐value = 0.126; Week 12, *p*‐value = 0.162; Table 4, Figure 4A–D).

**TABLE 4:** Average skin smoothness quantifications and average skin smoothness quantifications relative to baseline (Day 0; set at 100%).

Time point, number of participants (*n*)	Absolute number (AU)		Percent change from baseline (%)
	Average (SD)	*p*	Average (SD)
Baseline (Day 0), *n* = 13	1571.5 (752.7)		100.0
Day 1, *n* = 13	1398.0 (642.0)	0.059	90.1 (15.3)
Week 4, *n* = 13	1435.5 (701.9)	0.184	91.4 (23.9)
Week 8, *n* = 12	1492.7 (690.5)	0.126	90.7 (22.9)
Week 12, *n* = 13	1433.1 (661.0)	0.162	93.6 (22.7)

Abbreviations: AU, arbitrary unit; SD, standard deviation.

**FIGURE 5:**
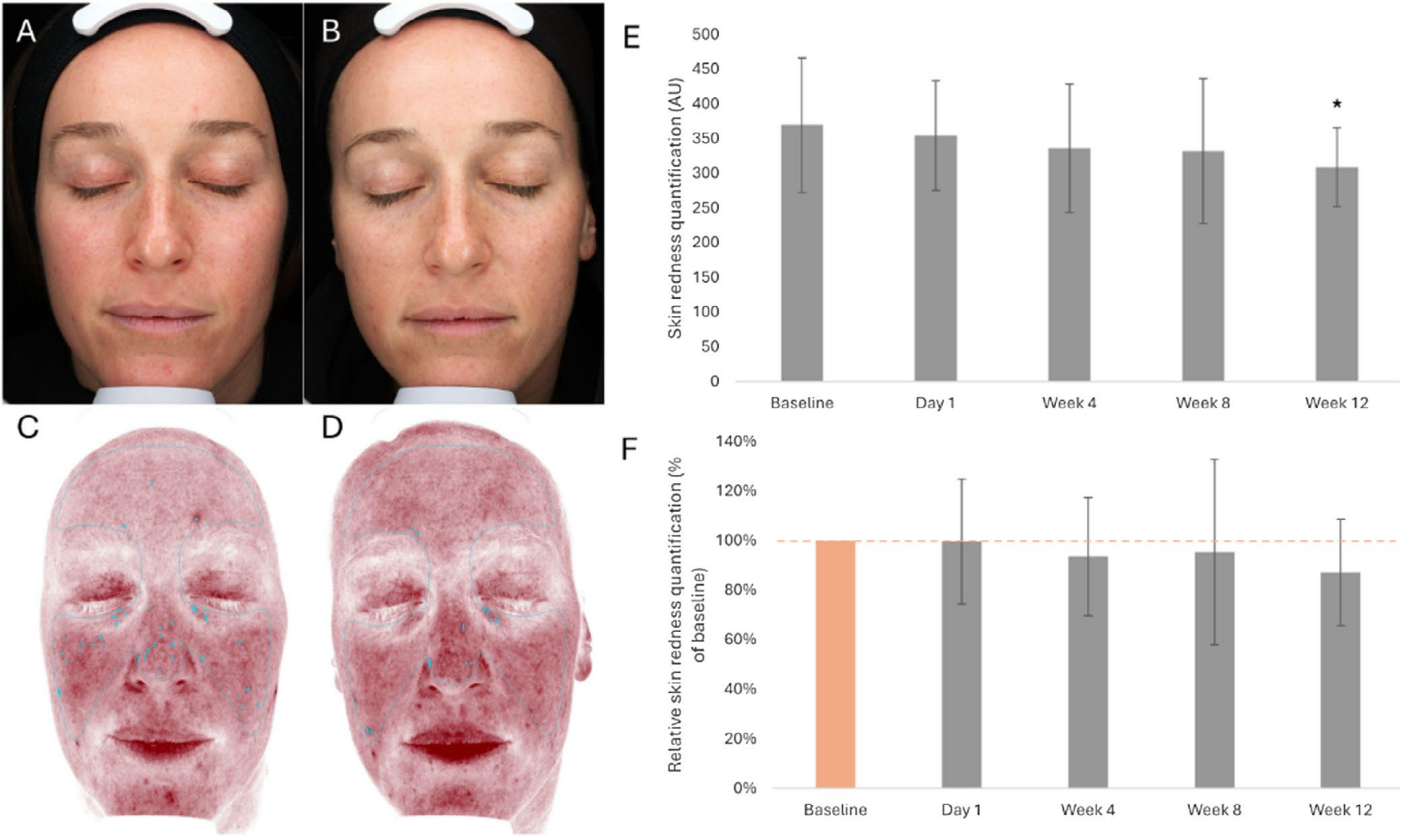
Skin redness. (A–D) Representative photographic images with standard lighting (A, B) and cross‐polarized light with VISIA “skin redness” filter (C, D), prior to the initiation of the treatment (Day 0; panels A and C) and at Week 12 after treatment initiation (panels B and D). (E) Average of skin redness quantifications prior to the initiation of the treatment (Baseline; Day 0), and at Day 1, Week 4, Week 8, and Week 12 after treatment initiation. (F) Average relative skin redness quantifications at baseline (Day 0; set at 100%) and Day 1, Week 4, Week 8, and Week 12 after treatment initiation. The error bars correspond to the standard deviation. *p‐value ≤ 0.05 is considered statistically significant. AU, arbitrary unit.

Average relative skin redness quantifications at Day 1, Week 4, and Week 8 remained within the 93.6%–99.6% range compared to the baseline condition (average relative value compared to baseline [set at 100%]: Day 1, 99.6%; Week 4, 93.6%; Week 8, 95.3%; Table 5, Figure 5A–D). No statistically significant differences between these conditions and the baseline condition were highlighted (Table 5, Figure 5A–D). Average relative value for the Week 12 condition was 87.2% of the baseline condition, with significant differences in the average skin redness quantifications obtained at this time point compared to the baseline condition (*p*‐value = 0.030; Table 5, Figure 5A–D).

**TABLE 5:** Average skin redness quantifications and average skin redness quantifications relative to baseline (Day 0; set at 100%).

Time point, number of participants (*n*)	Absolute number (AU)		Percent change from baseline (%)
	Average (SD)	*p*	Average (SD)
Baseline (Day 0), *n* = 14	369.5 (97.0)		100.0
Day 1, *n* = 14	354.5 (79.1)	0.660	99.6 (25.3)
Week 4, *n* = 14	336.2 (92.4)	0.315	93.6 (24.0)
Week 8, *n* = 13	332.5 (104.8)	0.295	95.3 (37.4)
Week 12, *n* = 14	308.5 (56.8)	0.030*	87.2 (21.5)

Abbreviations: AU, arbitrary unit; SD, standard deviation.

* *p*‐value ≤ 0.05 is considered statistically significant.

### 3.4 Participant Self‐Assessment

Based on responses to self‐assessment questionnaires, 56.3% of participants reported a satisfactory improvement in their acne at the conclusion of the study, with 75.0% of participants reporting some degree of improvement (slight to satisfactory) in severity of their acne. The average decrease in perceived acne severity was 29.7%. In addition, at the outset of the study, 68.8% of participants reported improvements in perceived pore size (i.e., less noticeable or smaller pores), 87.5% of participants reported improvements in skin texture (i.e., smoother skin). With regards to skin clarity, 50.0% of participants perceived a satisfactory or better improvement at the end of the study, and 87.5% of participants perceived some degree of improvement (slight to significant) in this parameter.

**FIGURE 6:**
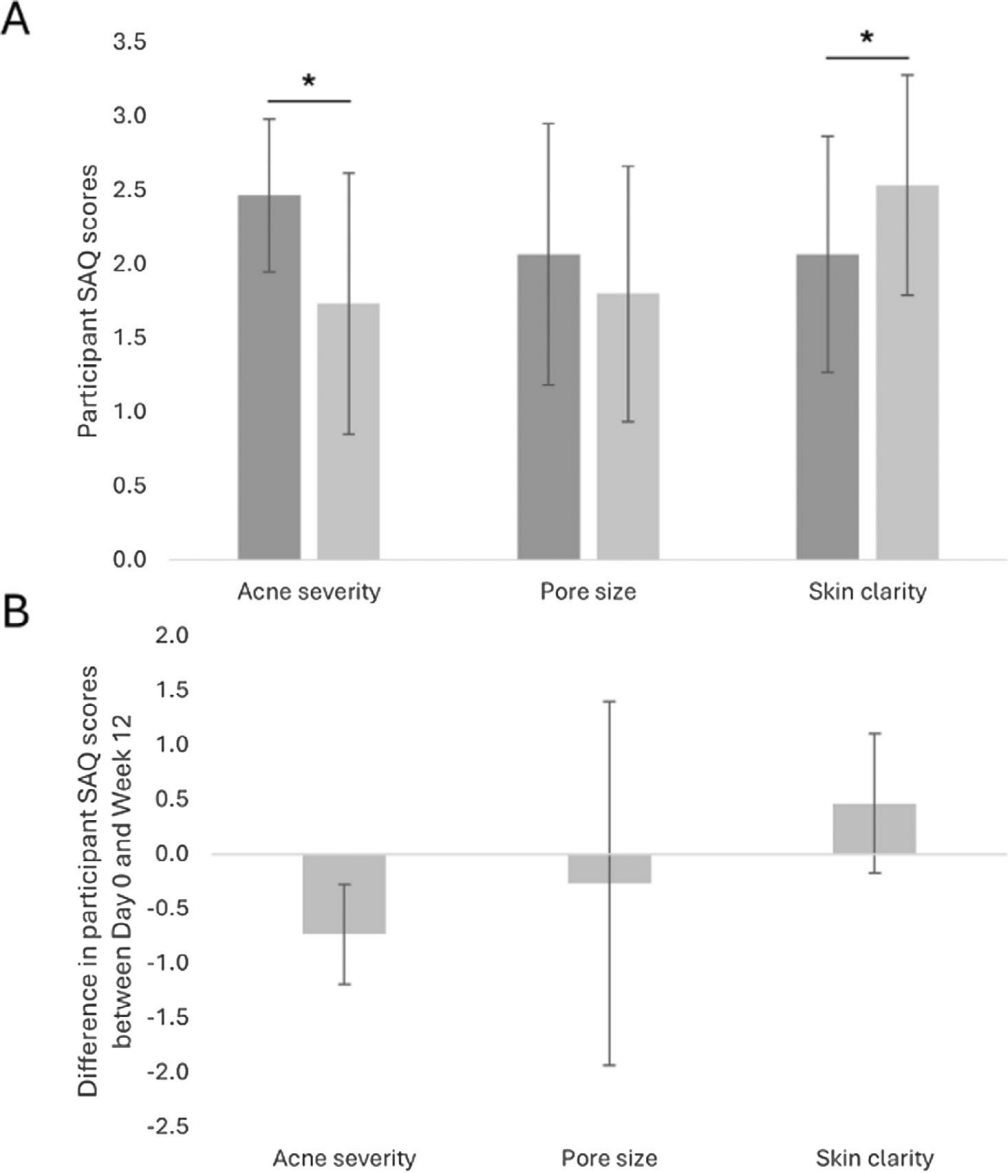
Participant self‐assessment of acne severity, pore size, and skin clarity. (A) Average self‐assessment scoring of acne severity, pore size, and skin clarity prior to the initiation of the treatment (Baseline; Day 0) and at Week 12 after treatment initiation. (B) Average relative change in self‐assessment scoring of acne severity, pore size, and skin clarity between baseline (Day 0) and Week 12. The error bars correspond to the standard deviation. *p‐value ≤ 0.05 is considered statistically significant. SAQ, self‐assessment questionnaires.

Further analysis of data retrieved from the self‐assessment questionnaires completed by the study participants highlighted decreases in average perceived acne severity scores and average perceived pore appearance at Week 12 compared to the baseline condition (Table 6, Figure 6). Indeed, average scores for perceived acne severity were 2.5 and 1.7 at baseline and Week 12, respectively, and average scores for perceived pore appearance were 2.1 and 1.8 at baseline and Week 12, respectively (Table 6, Figure 6A,B). Conversely, perceived skin clarity scores were higher at Week 12 compared to the baseline condition (2.1 at baseline vs. 2.5 at Week 12; Table 6, Figure 6). Average relative change in scores for these parameters was −0.7, −0.3, and 0.5, respectively, at Week 12 compared to the baseline condition (Table 6, Figure 6). Statistical analysis of these parameters highlighted significant differences in average perceived acne severity score (*p*‐value = 0.004) and average perceived skin clarity score (*p*‐value = 0.036) at Week 12 compared to the baseline condition (Table 6, Figure 6). Conversely, the difference in average perceived pore size between Week 12 and baseline was not statistically significant (*p*‐value = 0.689; Table 6, Figure 6A,B).

**TABLE 6:** Average self‐assessment scoring and average relative change in self‐assessment scoring between baseline (Day 0) and Week 12 for acne severity, pore size, and skin clarity.

Time point, number of participants (*n*)	Absolute number		Change from baseline
	Average (SD)	*p*	Average (SD)
Acne severity			
Baseline (Day 0), *n* = 15	2.5 (0.5)		
Week 12, *n* = 15	1.7 (0.9)	0.004*	−0.7 (0.5)
Pore size			
Baseline (Day 0), *n* = 15	2.1 (0.9)		
Week 12, *n* = 15	1.8 (0.9)	0.689	−0.3 (1.7)
Skin clarity			
Baseline (Day 0), *n* = 15	2.1 (0.8)		
Week 12, *n* = 15	2.5 (0.7)	0.036*	0.5 (0.6)

Abbreviation: SD, standard deviation.

* *p*‐value ≤ 0.05 is considered statistically significant.

## 4 Discussion

Acne vulgaris is a widespread skin disease affecting not only adolescents, but also adults, with potentially enduring mental and physical health consequences [1, 8, 9]. Evidence‐based guidelines support the use of both long‐term combination treatment and maintenance therapy for the management of acne [2, 18]. However, traditional therapies, such as topical and systemic treatments, can fail to achieve long‐term remission and may result in unwanted side effects [19]. Moreover, maintaining patient adherence to these long‐term treatments can be challenging [19]. For these reasons, it is equally important to make available more compact treatment modalities that are both safe and effective in reducing acne lesions and associated scarring.

Chemical peels offer an alternative therapeutic option for active acne and have been used as supplements to traditional acne treatments, as well as maintenance therapy after improvement or clearance of lesions [20]. Peels have been shown to reduce both inflammatory and non‐inflammatory acne lesions, though effects on more severe nodulocystic lesions were less pronounced [20, 21]. In addition, the safety and high tolerability of chemical peels have been shown consistently in studied populations [20].

The chemical peel investigated in this exploratory study is formulated with a synergistic blend of active ingredients to deeply cleanse, renew the skin, treat active breakouts, and improve overall texture (see composition in Table 7). Key components include: Salicylic Acid to help Unclogs pores, Lactic Acid to provide exfoliation, Resorcinol to reduce oil production. The peel is further enhanced by added biofunctional ingredients such as Licorice, to offer skin‐soothing benefits via its anti‐inflammatory action, Oleyl Adapalene to help manage acne lesions by regulating skin cell turnover and reducing inflammation, Saccharide Isomerate for strengthening the moisture barrier and to minimize water loss, and soothe sensitive, acne‐prone skin. Additionally, the antioxidant and anti‐inflammatory ingredient, Alpha Lipoic Acid combats acne‐causing bacteria and repairs damaged cells. Its action on scavenging free radicals, inhibiting inflammatory markers, and enhancing cellular metabolism leads to clearer skin, reduced pore size, and the gradual diminishing of scars over time [22].

**TABLE 7:** Shows the composition of the test chemical peel (in ranges only).

Ingredient	Wt%
Lactic acid	5–20
Salicylic acid	5–20
Resorcinol	0.1–20
Licorice extract	0.01–0.2
Alpha lipoic acid	0.01–0.2
Oleyl adapalate	0.1–0.4
Sachharide isomerate	0.2–1
Carrier (water and or ethanol)	Q.S to 100

Here, we report significant decreases in total acne lesions and papules following treatment with a commercially available chemical peel. Though trends towards decreasing numbers of pustules were also observed following the treatment, the difference with regard to baseline data was not statistically significant. Interestingly, previous studies with other chemical peels also reported higher response to treatment in papules than pustules [21, 23, 24].

Increased seborrhea has been clearly shown to be a major underlying cause of acne [2]. Previous studies have had mixed results regarding the effects of peels on sebum production [25, 26]. In the present study, while no significant changes in sebum production were observed during most of the study period, a relatively modest but statistically significant 6.38% decrease in sebum production was observed 12 weeks after the start of the treatment. Whether this decreased sebum production contributed to the reduced number of papules observed in the study remains to be established. Regardless, lower seborrhea remains a promising effect that is likely to benefit people receiving the treatment.

Improvements in skin appearance, specifically pore size and skin texture, were previously reported for chemical peels [24]. Here, we also observed improvement in pore appearance following the treatment. Conversely, no improvement in skin smoothness was evidenced in the present study.

Erythema or skin redness is a normal part of the healing process after chemical peeling; however, prolonged erythema is also one of the most common complications of the procedure [27]. In the present small‐scale study, participants exhibited no significant increases in skin redness. Instead, it appears the treatment procedure promoted moderate decreases in skin redness, with a statistically significant change observed 12 weeks after the treatment was initiated.

Finally, analysis of the subjective self assessments of treatment showed a relatively high proportion of participants reporting perceived improvements in acne severity and skin appearance.

Although this study provides valuable preliminary data regarding the benefits of the tested chemical peel treatment, several limitations must be acknowledged. Firstly, the study lacks a control group, which precludes the definitive conclusion that improvements in acne lesions and skin appearance are strictly associated with the treatment and not a natural progression of the disease. Furthermore, though Fitzpatrick skin types I–VI were represented, the study population was relatively small and homogenous, and results may therefore not be representative of other populations (e.g., people with more severe forms of acne, people from other age groups, people of other ethnicities). While the study effectively demonstrated the benefits of the novel peel, it is necessary to acknowledge the subjects' use of certain basic, necessary products. These included an inert cleanser, a basic moisturizer, and SPF‐30. The inclusion of these products was vital for managing skin sensitivity, which chemical peels can cause, especially when combined with routine activities like cleansing and sun exposure. We anticipate that these basic products had only a minimal impact on the final study results. Finally, the study period was also relatively short (12 weeks in total, with a follow‐up period of only 4 weeks after the third‐and‐final treatment administration), which limits the assessment of the potential longer‐term benefits of the treatment.

While the novel chemical peel evaluated here shows promise as a safe and gentle acne treatment requiring limited downtime for people receiving the treatment, additional studies on larger populations or different populations (e.g., different age groups), as well as assessments of other efficacy and safety parameters would be useful to support the benefits reported here. In addition, further research might consider the impact of combining various acids and bioactive ingredients in chemical peels on skin renewal and acne management, or the development of daily care products that may complement chemical peel treatment to promote skin health and limit acne relapse during inter‐treatment periods.

## 5 Conclusions

Chemical peels, such as the one evaluated in this preliminary study, present a safe and effective treatment choice for individuals suffering from mild‐to‐moderate acne. They can serve as a standalone option or be used alongside other topical or systemic therapies for managing acne and associated symptoms. While traditional treatments are still recommended for more severe or resistant forms of acne, this report highlights the potential role of chemical peels in a comprehensive management strategy. Nevertheless, large‐scale, controlled studies are needed to fully confirm these initial findings.

## Author Contributions

S.B. and S.R. designed the study. S.B. and A.C. were responsible for study execution and data collection. M.Z.H. was the study physician. M.V. performed statistical analysis of the data. S.R. analyzed the results and drafted the manuscript. All authors reviewed the manuscript and approved the final version. They take full responsibility for the content and writing of this article.

## Funding

This work was supported by Colgate‐Palmolive Company.

## Ethics Statement

The authors confirm that the ethical policies of the journal, as noted on the journal's author guidelines page, have been adhered to and the appropriate ethical review committee approval has been received. The study protocol was approved by the United States Institutional Review Boards (U.S. IRB) on the 3rd of October 2023. Informed consent, as well as permission of use of images taken during the study, were obtained from all study participants.

## Conflicts of Interest

All authors are employees of the Colgate‐Palmolive group of companies. The authors have no other conflicts of interest to declare.

## Supporting information


**File S1:** The inclusion and exclusion criteria for the study.

**File S2:** The self‐assessment questionnaires utilized by the study subjects to determine the qualitative effects of the tested chemical peel.

## Data Availability

The data that support the findings of this study are available from the corresponding author upon reasonable request.
