# Supplementary material for: Assessing the Benefits of an Innovative Chemical Peel Containing Biofunctionals on Adult Acne‐Prone Skin: An Exploratory Interventional Study: A Preliminary Report
Source: J Cosmet Dermatol. 2026 Mar 5;25(3):e70772. doi: 10.1111/jocd.70772 (PMC12962059; doi:10.1111/jocd.70772)
Supplement: Supplementary file 1 — File S1: The inclusion and exclusion criteria for the study. [file JOCD-25-e70772-s001.docx]

**Supplementary material S1. Study inclusion and exclusion criteria**

**Inclusion criteria**

- Evidence of mild to moderate acne, with moderate post-acne pigmentation, skin tone evenness and uneven skin texture
- Fitzpatrick skin types I-VI
- Age range from 25 to 40 years old
- No use of tanning beds or excessive sun exposure.
- Willingness not to use any undesignated products during the duration of the study
- Willingness to cooperate and participate by following study requirements as evidenced by reading/signing the Informed Consent Statement and the Panelist’s Instructions
- Willingness to refrain from smoking and vaping
- Willingness to use only products provided in the study
- Willingness to abstain from any other cosmetic procedures (e.g., injections, peels, lasers) in the area of treatment

**Exclusion criteria**

- Individuals with no visible acne, comedones, pustules, papules, cysts, or individuals with extensive uneven skin texture such as scarring
- Pregnancy or breastfeeding
- Inability to fulfill the requirements of the test
- Known allergies to any ingredient in the test product
- Systemic or cutaneous disease other than acne
- Treatment with systemic oral retinoids, oral contraceptives or topical acne treatments
- Anyone who has any dermatological (skin) disease(s), or any other acute or chronic diseases, other than acne
