# Supplementary material for: Assessing the Benefits of an Innovative Chemical Peel Containing Biofunctionals on Adult Acne‐Prone Skin: An Exploratory Interventional Study: A Preliminary Report
Source: J Cosmet Dermatol. 2026 Mar 5;25(3):e70772. doi: 10.1111/jocd.70772 (PMC12962059; doi:10.1111/jocd.70772)
Supplement: Supplementary file 2 — File S2: The self‐assessment questionnaires utilized by the study subjects to determine the qualitative effects of the tested chemical peel. [file JOCD-25-e70772-s002.docx]

**Supplementary material S2. Self-assessment questionnaires**

**Self-assessment questions collected at baseline (day 0):**

| **On a scale of 0 to 4, how would you rate the appearance of your acne?**  0 – None 1 – Minimal 2 – Mild 3 – Moderate 4 - Severe |
| --- |
| **On a scale of 0 to 4, how would you rate the oiliness of your skin ?**  0 – None 1 – Minimal 2 – Mild 3 – Moderate 4 - Severe |
| **On a scale of 0 to 4, how would you rate the appearance of your pore size?**  0 – None 1 – Minimal 2 – Mild 3 – Moderate 4 - Severe |
| **On a scale of 0 to 4, how would you rate the appearance of your discoloration? (dark spots and pigmentation)**  0 – None 1 – Minimal 2 – Mild 3 – Moderate 4 - Severe |
| **On a scale of 0 to 4, how would you rate the texture/smoothness of your skin? (visual or tactile)**  0 - Very smooth 1 - Moderately smooth 2 - Somewhat smooth 3 - Minimally smooth 4 - Uneven/not smooth |
| **On a scale of 0 to 4, how would you rate the overall evenness (clarity) of your skin tone?**  0 - Needs Significant improvement 1 - Needs Modest improvement 2 - Needs Slight improvement 3 - Needs minimal improvement 4 - Doesn't need improvement, looks great |

**Self-assessment questions collected at Week 12:**

| **On a scale of 0 to 4, how would you rate the overall improvement in your acne?**  0 – Worsening 1 - No change 2 - Slight improvement 3 - satisfactory improvement 4 - Significant improvement |
| --- |
| **On a scale of 0 to 4, how would you rate the improvement of oiliness of your skin?**  0 – Worsening 1 - No change 2 - Slight improvement 3 - satisfactory improvement 4 - Significant improvement |
| **On a scale of 0 to 4, how would you rate the improvement of pore size/appearance?**  0 – Worsening 1 - No change 2 - Slight improvement 3 - satisfactory improvement 4 - Significant improvement |
| **On a scale of 0 to 4, how would you rate the improvement in the appearance of scarring?**  0 – Worsening 1 - No change 2 - Slight improvement 3 - satisfactory improvement 4 - Significant improvement |
| **On a scale of 0 to 4, how would you rate the improvement in the appearance of your discoloration? (pigmentation)**  0 – Worsening 1 - No change 2 - Slight improvement 3 - satisfactory improvement 4 - Significant improvement |
| **On a scale of 0 to 4, how would you rate the improvement of texture/smoothness of your skin? (visual or tactile)**  0 – Worsening 1 - No change 2 - Slight improvement 3 - satisfactory improvement 4 - Significant improvement |
| **On a scale of 0 to 4, how would you rate the improvement of overall evenness (clarity) of your skin tone?**  0 – Worsening 1 - No change 2 - Slight improvement 3 - satisfactory improvement 4 - Significant improvement/looks great |
